# Coronary Intra-orbital Atherectomy Complications and Procedural Failure: Insight From the Manufacturer and User Facility Device Experience (MAUDE) Database

**DOI:** 10.7759/cureus.40817

**Published:** 2023-06-22

**Authors:** Rashid Alhusain, Dhruvil Patel, Heba Osman, Ahmed Subahi, Ahmed K Ahmed, Ahmed Shaikheldin, Sami Hussein, Ahmed Abdelrahim, Chaitu Dandu, Adam Chalek, Neel Patel, Mohamed Elhussein, Mohammad Hamza, Sardar Muhammad Alamzaib, Yasar Sattar, M. Chadi Alraies

**Affiliations:** 1 Internal Medicine, Detroit Medical Center, Detroit, USA; 2 Internal Medicine, Wayne State University School of Medicine, Detroit, USA; 3 Internal Medicine/Pediatrics, Wayne State University Detroit Medical Center, Detroit, USA; 4 Internal Medicine, Beaumont Hospital, Detroit, USA; 5 Research, Mayo Clinic, Jacksonville, USA; 6 Internal Medicine, Ascension Saint Joseph Hospital, Chicago, USA; 7 Vascular Surgery, Wayne State University School of Medicine, Detroit, USA; 8 Internal Medicine, New York Medical College/Landmark Medical Center, Woonsocket, USA; 9 Medicine, National Ribat University, Khartoum, SDN; 10 Internal Medicine, Albany Medical Centre, Albany, USA; 11 Cardiovascular Medicine, Marshall University Joan C. Edwards School of Medicine, Huntington, USA; 12 Internal Medicine, Icahn School of Medicine at Mount Sinai, New York, USA; 13 Cardiology, Detroit Medical Center, Detroit, USA

**Keywords:** maude, complications, interventional cardiology, orbital atherectomy, diamondback

## Abstract

Background: The Diamondback 360® Coronary Orbital Atherectomy System (Cardiovascular Systems Inc., St. Paul, MN) is the first and only orbital atherectomy system approved by the US FDA for the treatment of severely calcified lesions. While the device has proven to be safe in clinical trials, real-world data are minimal.

Methods: The Manufacturer and User Facility Device Experience (MAUDE) database was queried for reports on the Diamondback 360® Coronary from January 2019 to January 2022.

Results: A total of 566 events were reported during the study period. After the exclusion of duplicate reports, the final cohort included 547 reports. The most common mode of failure was break or separation of a device part (40.4%, n = 221) mainly due to breaking in the tip of the ViperWire (66.1%), driveshaft (22.7%), or crown (12.2%). The most common vessel associated with events was the left anterior descending artery (31.4%), followed by the right coronary artery (26.9%), left circumflex (21.6%), and left main coronary artery (6.4%). The most common clinical adverse outcome was perforation (33.0%, n = 181) with 23.7% resulting in cardiac tamponade. Most perforation cases were treated by covered stent (44.2%), surgery (30.5%), stent (98%), and balloon angioplasty (9%). There were 89 (16.3%) events of death with 67% due to perforation (p < 0.001).

Conclusion: Our study provided a glimpse of real-world adverse outcomes and common modes of failure due to orbital atherectomy. The most common mode of failure was the break or separation of a device part and the most common complication was perforation according to the MAUDE database. It will help physicians to anticipate complications and escalate care appropriately.

## Introduction

Heavily calcified coronary lesions during the percutaneous coronary intervention (PCI) can cause stent under-expansion and force malpositioning due to increased plaque resistance and tortuous architecture [1,2]. These features increase the risk of in-stent restenosis or thrombosis, which, along with heavily calcified lesions (independent risk factor itself), increase the risk for stent failure, repeat revascularization, and major adverse cardiac events (MACE) [3-6]. Despite drug-eluting stents lowering these risks, mortality remains a significant outcome in patients with severe coronary calcifications [7]. Furthermore, anywhere from 6% to 25% of patients with severe coronary artery disease, who need PCI, have heavily calcified vessels [6-8], posing a continued risk against favorable prognosis from intervention. To lower the risk of complications, modification of these heavily calcified plaques was introduced with a technique known as coronary atherectomy.

The Diamondback 360® Coronary Orbital Atherectomy System (Cardiovascular Systems Inc., St. Paul, MN) was designed as an adjunctive modifier for heavily calcified plaques. Approved by the FDA in 2013, it is the only orbital atherectomy system available in the USA [9]. It uses a rotating diamond-coated crown to shred coronary plaque by allowing bidirectional motion through a calcification. The tool works by the principles of differential sanding, where compliant native vascular tissue flexes away from the rotating crown while noncompliant plaque remains in its path and is rubbed off by the diamond-mounted crown; thereby improving the stent fit of the native coronary vasculature [10,11]. The ORBIT II trial, which was designed to evaluate the safety and efficacy of the orbital atherectomy system in treating severely calcified coronary lesions, validated the technology for the preparation of severe calcified coronary plaques. It showed a marked reduction in mortality, slow-flow or no-flow rates, and post-procedural cardiac complications [12]. Furthermore, the implementation of this technology has been evaluated to reduce the cost of performing PCI by more than $2,500 per procedure [13].

Due to these benefits, the Diamondback 360® Coronary Orbital Atherectomy System has seen almost a doubling in its use since approval in 2013 to 2016 as more complex cases are becoming manageable [14]. Simultaneously, complications including coronary perforation, coronary dissection, and in-procedure myocardial infarctions have also been reported [14,15]. Yet, there is a paucity of data associated with device-related complications and failure modes of this system. While the ECLIPSE trial aims to provide clinical evidence for orbital atherectomy use compared to conventional angioplasty for all plaque clearance [16], only one analysis by Khalid et al. of the Manufacturer and User Facility Device Experience (MAUDE) database from 2016 to 2018 describes the real-world complications of this system [17]. The purpose of this study is to identify features and report any complication trends of the Diamondback 360® Coronary Orbital Atherectomy System using post-marketing data from the MAUDE database that is published through the FDA since 2018 with continued interest to build on data for the real-world use of this system.

This article was previously presented as a meeting abstract at the 2022 Society for Cardiovascular Angiography & Interventions (SCAI) Meeting on May 21st, 2022.

## Materials and methods

Data source

The FDA created the MAUDE online database enlisting adverse events caused by approved medical devices. The MAUDE database contains reports submitted to the FDA by mandatory reporters (manufacturers, importers, and device user facilities) and voluntary reporters such as healthcare professionals, patients, and consumers [18]. The MAUDE database is publicly available and de-identified. Therefore, no institutional review board (IRB) approval was required for this study. We queried the database from January 2019 to January 2022 using the keyword “Diamondback Coronary.” The database was last accessed on February 18th, 2021, by two independent reviewers.

Outcomes and statistical analysis

The primary outcome of this study was the mechanisms of failure of the Diamondback coronary intra-orbital atherectomy system. Secondary outcomes included clinical consequences of device failure. The MAUDE database is unable to capture all the cases of turnpike utilization in the US. Hence, it cannot predict the actual incidence rate of failures or complications. Categorical variables were described as numbers, and all statistical calculations were performed with IBM SPSS Statistics for Windows, version 27.0 (IBM Corp., Armonk, NY) [19].

## Results

A total of 566 reports were found during the study period. After excluding duplicate reports (n = 19), our final cohort included 547 reports. The nature of the procedures and the clinical course of the patients were not described in all the reports (Table 1).

**Table 1:** Reports of the intra-orbital coronary atherectomy system failure in the MAUDE registry MAUDE: Manufacturer and User Facility Device Experience; RCA: right coronary artery; LAD: left anterior descending artery; LCX: left circumflex artery; LMCA: left main coronary artery.

Total number of events	547
Target vessel	
LAD, n (%)	172 (31.4%)
RCA, n (%)	147 (26.9%)
LCX, n (%)	118 (21.6%)
LMCA, n (%)	35 (6.4%)
Other, n (%)	14 (2.6%)
Insufficient/missing information, n (%)	70 (12.8%)
Failure method	
Break or separation, n (%)	221 (40.4%)
Entrapment, n (%)	44 (8.0%)
Noise/higher rate than expected, n (%)	9 (1.6%)
Flow lower than expected/not working, n (%)	3 (0.5%)
Insufficient information, n (%)	4 (0.7%)
None, n (%)	233 (42.6%)
Clinical consequence	
Perforation, n (%)	181 (33.0%)
Vessel dissection, n (%)	63 (11.5%)
Device embedded in tissue, n (%)	117 (21.3%)
Hematoma, n (%)	6 (1%)
Thrombosis/myocardial infarction, n (%)	13 (2.3%)
No reflow, n (%)	28 (5%)
Arrhythmias, n (%)	14 (2.5%)
None	76 (39.4%)
Patient outcome	
Death, n (%)	89 (16.3%)
No consequences, n (%)	167 (30.5%)
Recovered, n (%)	242 (44.2%)
Insufficient information, n (%)	49 (9.0%)

**Figure 1:**
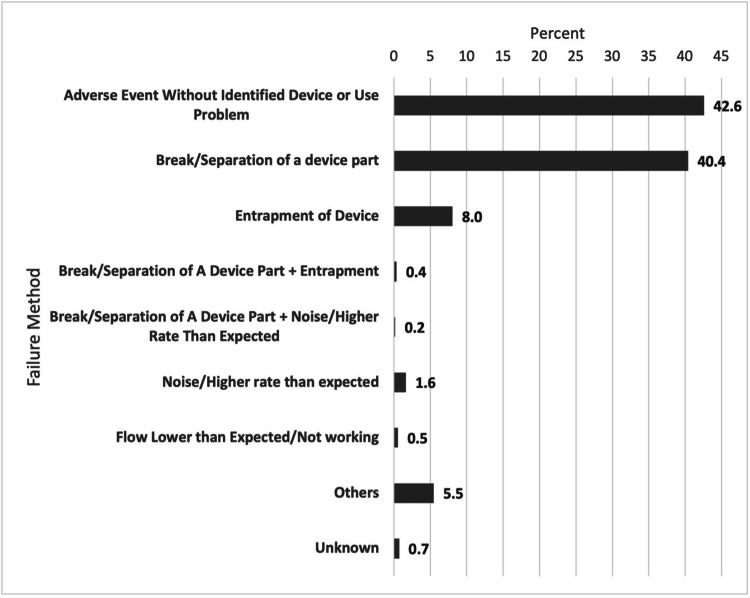
Percentage of each failure mode

The most common mode of failure was the break or separation of a device part (40.4%, n = 221) mainly due to breaking at the tip of the ViperWire (66.1%), driveshaft (22.7%), or crown (12.2%), followed by entrapment of the device (8%, n = 44) and noise/higher rate than expected (1.6%, n = 9) (Figure 1).

The most commonly observed clinical adverse outcome was perforation (33.0%, n = 181) with 23.7% resulting in cardiac tamponade. Most perforation cases were treated by a covered stent (44.2%), surgery (30.5%), stent (98%), and balloon angioplasty (9%). There were 89 deaths with 67% due to perforation (p < 0.001). Other common clinical adverse outcomes included foreign body embedment following breakage of the device (21.3%, n = 117), vessel dissection (11.5%, n = 63), no reflow following intervention (5%, n = 28), arrhythmias (2.5%, n = 14), myocardial infarction (2.4%, n = 13), and hematoma (1%, n = 6). Left anterior descending artery (LAD) was the most common vessel of intervention (31.4%, n = 172), followed by the right coronary artery (RCA) (26.9%, n = 147), left circumflex (LCX) (21.6%, n = 118), left main coronary artery (LMCA) (6.4%, n = 45), others (2.6%, n = 14), and unknown (12.8%, n = 70). LAD was significantly implicated in 47 events of death (52.8% of total death; p < 0.001) (Table 2).

**Table 2:** Patients’ outcomes categorized based on clinical consequence * Percentages within each row. ** Percentages within the total (547). *** Chi-square test was used.

Clinical consequence	Patient outcome*				Total**	P***
	No consequence	Recovered	Death	Unknown		
Perforation	3 (1.7%)	110 (60.8%)	60 (33.1%)	8 (4.4%)	181 (33.0%)	Less than 0.0001
Dissection	5 (7.9%)	47 (74.6%)	7 (11.1%)	4 (6.3%)	63 (11.5%)	Less than 0.0001
Hematoma	1 (16.7%)	4 (66.7%)	1 (16.7%)	0 (0%)	6 (1%)	0.646
Foreign body/device embedded in tissue	42 (35.9%)	56 (47.9%)	5 (4.3%)	14 (12.0%)	117 (21.3%)	0.001
Thrombosis/myocardial infarct	0 (0%)	5 (38.5%)	4 (30.8%)	4 (30.8%)	13 (2.3%)	0.005
No reflow	1 (%)	15 (%)	5 (%)	7 (%)	28 (5%)	0.001
Arrhythmias (including bradycardia)	1 (7.1%)	7 (50.0%)	6 (42.9%)	0 (0%)	14 (2.5%)	0.014

## Discussion

Our analysis of the MAUDE database showed two major categories of complications: (1) cardiac complications from device use, and (2) characteristic features of device failures. The results from both categories in our study provide insights into issues that operators must consider when preparing for and actively using the Diamondback 360® orbital atherectomy device. Our major findings are as follows: (a) the most common cardiovascular complication for this device was coronary perforation, which was the largest overall cause of mortality in our cohort. (b) Separation of the device’s components during the procedure was the largest category of device failures with ViperWire guidewire complications being the most reported malfunction.

In our cohort, the largest procedure-related event was coronary perforation. Analyses from the National Cardiovascular Data Registry CathPCI by Beohar et al. reported the incidence of coronary perforation increased with orbital atherectomy adaptability from 2009 to 2016 [14]. Multiple cohort studies analyzing in-procedural complications and outcomes of patients with orbital atherectomy have reported an incidence of perforation ranging from 1.8% to 3.0% [12,15,20]. Among the 547 reports analyzed in our study, 33.0% of cases experienced coronary perforation, which consequently was the largest cause of mortality events too. The likelihood of perforation during PCI was found to increase when severe coronary calcifications were present [11,21], which are the exact conditions under which the Diamondback 360® Coronary Orbital Atherectomy System is indicated for. Plaque presence lowers coronary vessel compliance as the cross-sectional area grows [22]. The Diamondback coronary atherectomy system device generates a differential sanding effect that relies on the compliance of healthy coronary walls to flex out of the path of the device's rotating crown. With increased calcifications, the ability for native walls to flex is reduced, resulting in potentially increased native wall surface area having contact with the device, potentially damaging these regions, and predisposing to perforation. Manufacturer recommendations note that experiencing resistance during plaque clearance is a likely cause of vessel perforation [11]. Thereby operators should use their best judgment when experiencing resistance or difficulty navigating during this device operation and be prepared to initiate any required intervention for perforation management.

The management of coronary perforation with covered stents was the most frequent strategy to manage this complication in our cohort. Covered stents are being used to prevent surgical intervention in coronary perforation but unfortunately do not have a high success rate with 53% of coronary perforations treated with covered stents by Lemmert et al. eventually still requiring pericardiocentesis due to persistent leaks [23,24]. They are also associated with an increased rate of stent thrombosis in a multi-year follow-up by Ford et al. [25]. Therefore, preparation for these complications should still be considered in patients undergoing orbital atherectomy, including a multidisciplinary cardiac and surgical team ready for possible surgical intervention, which was the second most common intervention for perforation in our cohort.

We also found among the failure reports that 40% of the cases were due to physical device damage during the procedure. The most common complication was the destruction of the ViperWire guidewire that was made specifically for the Diamondback 360® Coronary Orbital Atherectomy System. Manufacturer protocols note that using the wire with bends, kinks, or loops when operating the device for more than five minutes at a time can predispose the wire toward these complications. Checking the wire prior to implantation and cautiously feeling for these features as the device catheter is threaded through the guidewire is recommended. While there are no reports describing the management of ViperWire destruction in the coronary orbital atherectomy system, operators should carefully remove the device wire and open a new device package.

Guidewire bias occurs when the wire is preferentially oriented to one side of the vessel rather than the center of the plaque during insertion, resulting in potentially increased damage to that side during device use [26]. This feature was reported by Hayashi et al. when using ViperWire, which is predisposed to pseudoaneurysm formation [27]. This feature can predispose to perforation and the risk of this incidence continues to increase as vessel architecture becomes more complex in its course. In such cases, the differential sanding phenomena may over-flex compliant vessels, causing more damage to native coronary walls without adequately removing the plaque burden. Operators must be aware of the wire positioning relative to the plaque and coronary walls prior to initiating device movement through a vessel to prevent such incidents.

Fractures and dislodgement of the device crown have also been reported as complications of the Diamondback 360® orbital atherectomy system [28-31]. Most commonly, the crown became fully detached and would remain within the target vessel. Often this would occur after multiple passes of the device through stenosed arteries with increasing crown rotational speed [17]. Significant tortuosity and uneven plaque morphology have been found to increase the risk of crown fracture. Furthermore, to target these plaques, more bidirectional passes are required, yet the crown detachment risk significantly increases after 20 passes within five minutes, according to the manufacturer [11,29]. Therefore, operators must balance lumen clearance expectations with these device limits to prevent crown-related complications. Removal of the broken crown was successfully done with the use of a gooseneck snare over a newly inserted guidewire catheter in one case or using a tapered microcatheter to capture the tip over the same ViperWire as a device in another case [28,29]. Shlofmitz et al. also provide significant instruction on device techniques to minimize the incidence of crown complications. Using the same speed in each direction of plaque clearance with a slow and steady advancement was recommended to lower the risk of crown detachment while maximizing plaque clearance diameter [32]. Further, Sotomi et al. show that the advancement of the device at lower speeds (1 mm/s compared to 10 mm/s) irrespective of crown rotational speed resulted in more plaque clearance [33]. Therefore, operator technique can be a significant factor in preventing device-related complications and must be properly reviewed.

LAD, followed by the RCA interventions, was responsible for the greatest proportion of complications in our analysis. In a retrospective cohort investigation of plaque distribution within 309 patients by Wasilewski et al., the greatest calcification burden was observed in the proximal LAD followed by the RCA. This was attributed to both the LAD and RCA being exposed to greater hemodynamic wall shear stress than other coronary arteries, attributed to these vessels' uniform morphology and proximity to cardiac output. This stress creates injuries that produce inflammation and increase the risk of calcification formation [34]. The likelihood of noncompliance due to severe plaque burden is highest in these arteries, which most likely contributed to the increased complication rates. Operators must be wary of these features when intervening in both the LAD and RCA as these are very common targets for PCI.

## Conclusions

Despite clinical trials demonstrating the safety of the intra-orbital atherectomy coronary system, complications can still occur. These databases (e.g. MAUDE) serve to inform operators about potential risks and complications associated with the use of the device. Physicians should be aware of potential complications and escalate care appropriately, especially in cases involving a perforation.
